# The Toothbrushing Boost Toolbox: competence-building behavioral strategies for oral health

**DOI:** 10.3389/fpubh.2026.1834743

**Published:** 2026-07-15

**Authors:** Shalya Anand, Maria Almudena Claassen, Christine A. Riedy, Ralph Hertwig

**Affiliations:** 1 Center for Adaptive Rationality, Max Planck Institute for Human Development, Berlin, Germany; 2 Department of Oral Health Policy and Epidemiology, Harvard School of Dental Medicine, Boston, MA, United States

**Keywords:** behavior change, behavioral science, boosting, health promotion, nudging, oral health, public health

## Abstract

Oral disease is the most prevalent noncommunicable disease globally, affecting an estimated 3.7 billion people and imposing an annual economic burden of roughly US$710 billion—even though preventive recommendations for oral health are clear and widely known. This striking gap between knowledge and behavior points to a need for complementary approaches that help people translate awareness into action. Despite this need, behavioral science is an underexamined approach in oral health: Our bibliometric review found only 360 behavioral science publications addressing oral health compared with thousands in other domains, and most oral health studies were non-experimental. We argue that boosting, an approach from behavioral science that builds competences and respects autonomy, is a practical complement to traditional oral health education. We introduce the Toothbrushing Boost Toolbox: a set of simple, memorable strategies that map onto common behavioral barriers. These boosts are low-cost, adaptable across age groups and literacy levels, and could be integrated into clinical practice, public health programs, and digital tools. We further argue that boosting should be embedded within a broader agenda of system-level reform, including universal access and subsidized preventive care, and propose practical steps for implementing, evaluating, and co-designing boost interventions. An oral health agenda that is informed by behavioral science can help shift oral health promotion from providing information to fostering competence and sustained daily practice.

## 1 Introduction: rethinking strategies for promoting oral health

There is no health without oral health (1). Healthy ageing is more than the mere absence of disease—it involves fostering and sustaining the functional abilities that promote well-being, happiness, and fulfilment (2, 3). A central, often underappreciated contributor to healthy ageing is good oral health, defined as “the ability to speak, smile, smell, taste, touch, chew, swallow, and convey a range of emotions through facial expressions with confidence and without pain, discomfort, and disease of the craniofacial complex” (4) p. 229. Oral health is a key determinant of overall health, nutrition, social interaction, and quality of life (2, 4, 5). The most common oral diseases—dental caries, periodontal disease, and tooth loss—manifest in pain, tooth sensitivity, inflamed gums, or halitosis, and can drive systemic inflammation that exacerbates cardiovascular disease, diabetes, and neurodegenerative disorders (6–8). Yet despite being largely preventable, oral disease is the most prevalent noncommunicable disease worldwide, affecting 3.7 billion people [compared to 522 million people with cardiovascular diseases and 86 million with cancer (9)] and producing an estimated global economic burden of over US$710 billion annually (10).

Although many oral health promotion strategies rightly focus on awareness and access to oral healthcare, these measures alone have limited potential to produce sustainable behavior change. Knowledge, even combined with intention, is no guarantee of action. Examples of this intention–behavior gap (11) include being too tired to brush one’s teeth at night, or too rushed to brush properly in the morning. And some seemingly healthy actions can ultimately be detrimental. For instance, excessive use of antiseptic mouthwash may disrupt the oral microbiome (12). Furthermore, while educational programs such as motivational interviewing (a collaborative patient-centered interaction) (13) achieve short-term success in sharing knowledge and promoting isolated behaviors, the effects of most programs attenuate over time (14–16). Current programs generally do not address the behavioral drivers that determine everyday oral health practices: Choices about when and how to brush, whether to floss, how much sugar to consume, and how often to visit the dentist are shaped by habits, motivational drivers, affective states, cognitive constraints, and environmental cues (17, 18).

The current clinical–biomedical paradigm should therefore be complemented with behavioral science-informed interventions that strengthen people’s competences to make and sustain healthier oral health routines. Approaches from the behavioral and social sciences are not well-integrated into oral health prevention and promotion (17), and calls to embed these approaches within research and clinical practice continue to grow (18, 19). Although some interventions have begun to draw on behavioral theories, uptake remains limited (20, 21), and meta-analytic evidence suggests weak effects (22, 23).

Boosting is a promising approach from behavioral science that aims at increasing existing or instilling new competences. Boosts are competence-building interventions or decision tools that strengthen people’s skills, knowledge, behaviors, or ability to reshape their external environment (24). Unlike nudges, which encourage specific behaviors by altering the choice architecture (25), boosts aim to be durable and to respect people’s autonomy by explicitly building competences and decision-making skills, giving people the ability to act in accordance with their own personal goals (26, 27). Boosting is rooted in the concept of bounded (also known as adaptive) rationality: People act adaptively in the context of constraints and equipping them with simple strategies and competences allows them to navigate those constraints more effectively (28, 29). Boosting can be seen as operationalizing the action-implementation layer of established health-behavior frameworks [e.g., HBM - Health Belief Model; COM-B - Capability, Opportunity, Motivation Behavior system; HAPA - Health Action Process Approach) (30–32)] and translating constructs from health-behavior theory, such as cues to action, capability, and volitional planning, into concrete, autonomy-preserving strategies that individuals can deploy in everyday routines. Competence-enhancing boosts such as quick rules, implementation intentions, and self-nudges have been shown to improve dietary choices (33, 34).

Here we first highlight the research gap in behavioral science within oral health and propose boosting as a novel approach to support oral health behavior change. We introduce the Toothbrushing Boost Toolbox, an actionable evidence-based set of strategies to be empirically validated in oral health settings, that can be integrated into oral health promotion initiatives and embedded within public health campaigns and policy frameworks. Because toothbrushing is a frequent and potentially highly routinized behavior, even small improvements in consistency or technique may compound into substantial long-term gains in oral and general health.

## 2 The research gap

Behavioral science research has paid considerably less attention to oral health than to other health domains. Indeed, in their examination of nudging interventions on oral health behaviors, Kazemian et al. (35) had to draw on interventions from other health-related areas (e.g., dietary choices, tobacco and alcohol cessation). This relative lack of studies integrating behavioral science and oral health was confirmed by our own bibliometric analysis, which examined the number of publications involving behavioral science in several common health domains: physical activity, dietary behavior, smoking, alcohol consumption, medication compliance, oral health, risky sexual behavior, and sleep (36).

**Figure 1:**
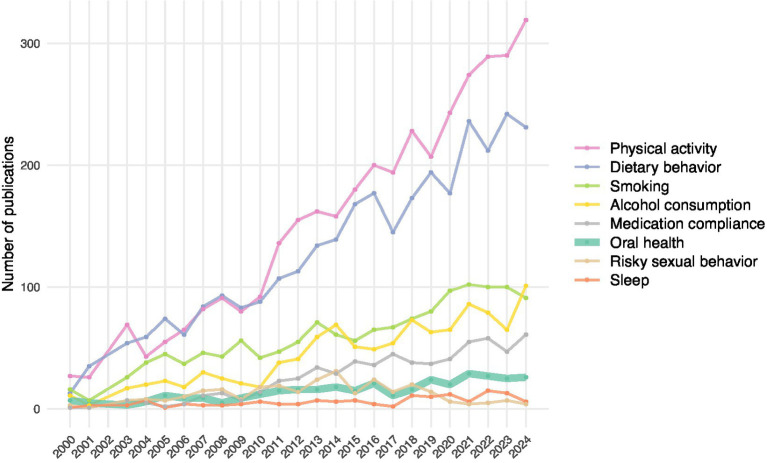
Behavioral science publications by health domain (2000–2024). Number of publications is drawn from Scopus-indexed articles and reviews in the English language, from TITLE-ABS-KEY searches, combining domain-specific and behavioral science terms. See Supplementary material for full details on search strings.

Figure 1 and Box 1 show that between 2000 and 2024, only 360 publications addressed behavioral science in oral health—not even 10% of the 3,942 publications on physical activity. Moreover, while research interest in behaviors such as physical activity and nutrition has grown substantially over the past two decades, studies on oral health (as well as risky sexual behaviors and sleep) have remained consistently low. Of the existing 360 oral health publications, the vast majority are non-experimental (87%), and fewer than 10% (*n* = 27) are randomized controlled trials that vary widely in participant populations, outcomes measured (e.g., psychological, behavioral, clinical), and the extent to which behavioral theory informs intervention design (see Online Materials for a list of studies), suggesting that the field has yet to fully harness what behavioral science has to offer.

BOX 1 **Oral health environments as the ideal environment to study health behavior change**

A secondary but compelling insight from our bibliometric analysis is that oral health research is a uniquely suitable context for studying health behavior. First, oral health behaviors—such as brushing, flossing, or using mouthwash—are simple, discrete, and easily observable, unlike more complex health behaviors such as changing one’s diet. Second, clinical outcomes can be measured with objective, quantifiable indicators such as the dental plaque index, the Gingival Bleeding Index, or the Visible Plaque Index (26, 41). Third, most (85%) of the identified studies took place in controlled clinical environments such as dental offices, which are highly suitable for standardized interventions and measurement. Fourth, the routine nature of dental check-ups enables longitudinal follow-up; in 92% of the identified studies, researchers were able to track the sustainability of behavior change over time. Finally, oral health studies frequently involve diverse populations—including children, older adults, patients with medical conditions, and individuals across diverse geographical areas and socioeconomic backgrounds—and can therefore provide insights that are more globally representative than those found, for instance, in psychological science (42) (see Supplementary material for details).

Several studies directly illustrate the promise of behavioral science approaches in oral health. Holloway et al. (37) and Dimenäs et al. (38) found that brief, dentist-delivered interventions centered on goal setting and planning produced significantly greater reductions in clinical outcomes, including gingival bleeding, compared to standard education. Schüz et al. (39) demonstrated that a brief planning intervention relying on implementation intentions (40) (i.e., planning when, where, and how to floss) increased flossing frequency at 2 and 8 weeks, with the strongest and most durable effects found in people who had already intended to floss. Trials such as these indicate that simple behavioral strategies can meaningfully improve oral health behavior.

## 3 Boosting decision-making in oral health

Boosts equip individuals with simple decision strategies. For instance, in the Dutch Reach boost, people learn to open a car door with their far hand. This single competence can help prevent them from hitting cyclists when they open the door: Because they turn their body, they are more likely to notice passing cyclists (43). Another boost, the 20–20–20 rule, helps people reduce digital eye strain by taking a 20-s break to look at something 20 feet away every 20 min (44). Building on examples like these, we developed the Toothbrushing Boost Toolbox (Table 1). We followed three steps to do this: First, we drew categories of behavioral challenges from the challenges to successful health behavior change identified by Rothman et al. (45). Second, we identified actionable strategies as evidence-based oral-hygiene targets (brushing frequency, duration, technique; post-brushing behavior; adjunctive) from the clinical literature [(46, 47); see Supplementary Table S1]. Third, we matched each challenge to a boost linked to a behavioral mechanism with documented effects on health behavior (e.g., implementation intentions, habit stacking, mindful action). This list is not exhaustive; its purpose is to highlight major behavioral barriers and illustrate how they can be matched to potential boosts.

**Table 1:** The Toothbrushing Boost Toolbox.

Challenge to behavior change	Behavioral mechanism	Boost	Actionable strategy
Insufficient motivation	Temptation bundling. Pairs an immediately rewarding activity with a beneficial but effortful behavior to increase adherence (59).	First brush, then fun	Make toothbrushing the ticket to a pleasurable activity (e.g., “no checking my phone until I brush”).
Failure to turn intention into action	Implementation intentions. If–then planning links cues to actions and increases the likelihood of performing the intended behavior (52, 60).	Brushing anchor	Couple toothbrushing to a specific existing cue (e.g., immediately after waking up or before leaving the bathroom).
	Self-nudging/choice architecture. Changing the immediate environment to increase cue salience and reduce friction supports action initiation (25, 27).	Visible brushing tools	Keep interdental brushes, a tongue scraper, or other tools in a visible, easy-to-reach place next to the toothbrush to cue daily use.
	Habit stacking. Pairs a new behavior with an existing habit to exploit the cue-response architecture; also called piggybacking (53, 61).	First ___, then ___	Stack toothbrushing onto an existing daily habit using a personally meaningful script, such as “First meds, then mouth,” for people who take medication, or “First teeth, then toys” for children’s routines.
Old habits interfere with a new plan of action	Overriding habitual behavior through sensory reframing/cognitive reappraisal. Reinterpret sensory feedback as a positive cue to support the behavior (62).	Spit, do not rinse	After brushing, spit but do not rinse with water; treat the residual minty film as fluoride protection.
New behavior is not sustained	Task chunking/proceduralizing. Breaks a complex task into an ordered sequence, reducing cognitive load and improving thoroughness (63, 64).	Four-corner brushing	Divide your mouth into four quadrants and brush ~30 s per quadrant to reach 2 min in total.
	Mindfulness. Present-moment attention increases focus and may support more thorough toothbrushing practices; it may also positively affect motivational processes and learning (50, 65).	Mindful brushing boost	Brush mindfully: Focus on the sensation of the bristles making small circular motions on all tooth surfaces. Avoid distractions such as phones while brushing.

The boosts are short, memorable, and repeatable actions that can simplify brushing, improve technique, and support the development of automaticity in oral health behaviors (48). Their goal is not only improving behavior in the moment, but also to help to proceduralize it over time. While these boosts have yet to be empirically validated in oral health behaviors, these techniques have shown promise in adjacent health domains. By promoting habit formation, they reduce reliance on conscious effort, momentary cues, and fluctuating motivation: Once a behavior becomes embedded in a stable routine, it is more likely to persist even under everyday constraints such as fatigue, distraction, or time pressure (49). The mindful brushing boost, which asks people to focus on the activity of brushing, may be especially useful because it can strengthen procedural learning and may support motivational processes such as intrinsic engagement and self-efficacy that are relevant to sustained behavior change (50). Mindfulness training has, for example, produced significant reductions in smoking in a randomized trial (51). Importantly, many of these boosts are also highly personalizable. Implementation intentions (i.e., planning when, where and how to perform an action) can be linked to whichever cue in a person’s routine is already stable and meaningful, and have shown robust effects across different health behaviors (52). Similarly, habit-stacking prompts can be adapted into personally resonant scripts (e.g., “First __, then teeth”), and have been associated with successful flossing habit formation (53), and environmental supports (e.g., a timer, favorite song, or Toothbrushing app) can be tailored to an individual’s context. Lastly, compared to more intensive educational programs, boosts are low-cost, easy to communicate, and feasible to implement and adapt across age groups, literacy levels, and cultural contexts.

## 4 A call to action: building a behavioral science-informed oral health agenda

The Consensus Statement (19) calls for integrating behavioral science into oral health education, research, and service delivery; adopting mixed-methods and community-engaged approaches; developing culturally adapted, theory-based interventions; and ensuring equitable inclusion of underserved populations. With the Toothbrushing Boost Toolbox we want to respond to this call, by offering a first concept of how a set of practical, skill-focused strategies that are adaptable across multiple settings—primary care, community outreach, schools, and digital platforms—and across population groups, from adolescents forming lifelong habits to older adults managing chronic conditions, can be used to build sustained oral health routines. By equipping individuals, caregivers, and frontline staff with simple heuristics and habit-forming routines, the toolbox aims to turn awareness into sustained practice. At the same time, these Toothbrushing boosts should be understood as a preliminary step towards building a comprehensive toolbox of boosts for dental health which we are currently developing and testing in the field. Future work should extend our approach to other dimensions of oral health behavior, including sugar reduction, dental attendance, denture care, oral self-examination, smoking-related oral health risks, and care routines for dependent populations.

Boosting is not a cure-all. Structural barriers such as cost, challenges in healthcare provision, and the broader social determinants of oral health limit the reach and effectiveness of any individual-level intervention. Consequently, boosts must be embedded within a broader, system-level agenda that secures universal access to preventive care, affordable hygiene products, and integrated oral health services. Behavioral competence-building and system reform are complementary—both are valuable to advance population oral health and reduce oral health inequities worldwide.

We suggest five main practical recommendations for integrating the Toothbrushing Boost Toolbox into the oral healthcare system:

- Evaluate effectiveness of boosts in feasibility studies with behavioral and clinical outcomes (e.g., tooth brushing frequency, Plaque Index, Gingival Bleeding Index) and acceptability and usability across relevant demographic groups.
- Develop and publicly disseminate simple, evidence-based toolboxes that translate boosts into action and simple practice. Existing examples include memory aids for oral healthcare advice (54), oral health toolkits for dental teams (55), and community-outreach oral health resources (56).
- Use implementation science to guide the scale-up, adaptation, and evaluation of boosting strategies from the Toothbrushing Boost Toolbox over time, analyzing their effectiveness, uptake, feasibility, and sustainability in real-world dental settings (57).
- Co-design boosting interventions with end users, caregivers, and community stakeholders to ensure cultural relevance, acceptability, and equity (e.g., through participatory design approaches and community partnerships) (58).
- Tailor and personalize boosts for diverse populations: Older adults may benefit from cues linked to medication routines; children may respond better to playful scripts and songs; people with disabilities may prefer tactile or voice-based cues; and low-literacy communities may benefit from visual or oral storytelling.

## 5 Conclusion: from awareness to empowerment

Oral health is more than a clinical issue—it is a behavioral, psychological, and social challenge that demands a reimagined, evidence-informed response with strategies rooted in the way people think and live. Embracing behavioral science and empowering individuals through boosting can close the gap between awareness and action and advance oral health and health equity. Boosts offer practical rules, simple decision heuristics, skills for reshaping one’s immediate environment, and the self-efficacy to sustain healthy routines. They respect people’s autonomy while building their competences, thereby helping to bridge the gap between knowing and doing. Behavioral science should no longer be brushed aside in the quest for oral health—it is time to empower people to smile with agency.

## Data Availability

The datasets presented in this study can be found in online repositories. The names of the repository/repositories and accession number(s) can be found at: https://osf.io/vbtqy/overview?view_only=4dffc417b80c4b458285f2b3c331b206.
