# Supplementary material for: The Toothbrushing Boost Toolbox: competence-building behavioral strategies for oral health
Source: Front Public Health. 2026 Jul 15;14:1834743. doi: 10.3389/fpubh.2026.1834743 (PMC13416245; doi:10.3389/fpubh.2026.1834743)
Supplement: Supplementary file 1 [file Table_1.DOCX]

**Supplementary Materials for *The Toothbrushing Boost Toolbox: Competence-Building Behavioral Strategies for Oral Health Promotion***

Additional material can be found on the OSF: <https://osf.io/vbtqy/overview?view_only=4dffc417b80c4b458285f2b3c331b206>

1. **The research gap: Bibliometric review**

**Search strings used for bibliometric search in Scopus**

Search strategy and inclusion criteria. The following search string was used to search articles in Scopus for each domain: TITLE-ABS-KEY (…..[specific domain terms]….) AND TITLE-ABS-KEY ("behavioral science" OR "behavioral psychology" OR "behavioral insights" OR "decision science" OR "behavioral interventions" OR "behavioral mechanisms" OR "behavioral strategies" ) AND PUBYEAR > 1999 AND PUBYEAR < 2025 AND ( LIMIT-TO ( DOCTYPE,"ar" ) OR LIMIT-TO ( DOCTYPE,"re" ) ) AND ( LIMIT-TO ( LANGUAGE,"English" ) )" )

The specific search terms for each domain were the following. For **oral health**: "oral health" OR "dental health" OR "dentistry”. For **dietary behavior**: nutrition OR eating OR diet OR "food intake”. For **sleeping**: “sleeping” or “sleep behavior”. For **physical activity**: "physical activity" OR exercise. For **medication compliance**: “medication adherence” OR “medication compliance”. For **smoking**: smoking OR tobacco OR "smoking cessation”. For **alcohol consumption**: "alcohol consumption" OR "alcohol use" OR "binge drinking”. For **risky sexual behavior**: “sexual risk behavior” OR “unprotected sex” OR “condomless sex”.

The **folder Figure1** contains .csv files with the identified papers for each domain, as well as an *R* script to create the figure.

See **Oral_health_papers.csv** for the full list of 360 oral health publications identified and **RCTs.csv** for a list of the randomized controlled trials. The *R* script **classification_results.R** provides an overview of the article types and study design of the 360 publications.

1. **Boosting Decision-Making in Oral Health: The Toothbrushing Boosting Toolbox**

**Table S1. Summary of Evidence-Based Oral Hygiene Behaviors**

|  | **Target Behavior** | **Recommendation** | **Key Evidence** |
| --- | --- | --- | --- |
| Brushing | Frequency | Brush twice a day | Brushing twice daily is a foundational recommendation for effective plaque removal and the primary prevention of gingivitis (Chapple et al., 2015). |
|  | Timing | Brush right after eating | There is no significant difference in enamel wear between immediate and delayed brushing. To maximize the caries-preventive effect of fluoride by promptly clearing food, brushing right after eating is may be recommended (Fernández et al., 2024; Hong et al., 2020). |
|  | Duration | Brush for 2 minutes | Brushing for 2 minutes removes significantly more plaque than brushing for 1 minute (Steuntjes et al., 2023).The longer the brushing the better (Gallagher et al., 2009). |
|  |  | Spit, don’t rinse | Spitting out excess toothpaste and not rinsing with water increases fluoride retention in the oral cavity (Parakaw et al., 2024) |
| Tongue Cleaning |  | Clean the tongue daily as part of the oral hygiene routine | Tongue cleaning, particularly with a scraper, is effective in reducing tongue coating, levels of oral bacteria, and halitosis compared to brushing alone (Kuo, Y. W., Yen, M., Fetzer, S., & Lee, J. D. (2013); van der Sleen et al., 2010)  Muthu et al., 2021; Pedrazzi et al., 2020). |
| Interdental Cleaning |  | Clean between teeth daily | Interdental brushes are superior to dental floss for reducing plaque and gingival bleeding (Kotsakis et al., 2021) and to treat gingivitis (Worthington et al., 2019). Self-performed flossing has weak benefits, but can be used when interdental brushes do not fit (Bercier et al., 2008; Chapple et al., 2015). |
| Mouthwash |  | Consider as an adjunct to mechanical hygiene when appropriate | Essential oil and Cetylpyridinium Chloride (CPC) mouthwashes provide a statistically significant additional reduction in plaque and gingivitis (Haps et al., 2008) but frequency concerns should be considered due to potential negative effects on the oral microbiome (Brooks et al., 2023). |
